# SpineCor treatment for Juvenile Idiopathic Scoliosis: SOSORT award 2010 winner

**DOI:** 10.1186/1748-7161-5-25

**Published:** 2010-11-10

**Authors:** Christine Coillard, Alin B Circo, Charles H Rivard

**Affiliations:** 1 Sainte Justine Hospital, 3175, Chemin de la Côte-Sainte-Catherine Montréal, H3T 1C5 Quebec, Canada; 2 University of Montréal, 2900, boul. Édouard-Montpetit Montréal (Québec) H3T 1J4, Canada

## Abstract

**Introduction:**

Juvenile idiopathic scoliosis is a condition used to describe patients who are least 4 years of age but younger than 10 when the deformity is first identified. In these patients, the condition is usually progressive and those that are diagnosed at five years or younger have a high chance of progression to a large curve, with additional pulmonary and cardiac complications. The main form of conservative treatment for juvenile scoliosis is the use of a bracing system. This prospective interventional study was conducted to evaluate the effectiveness of the Dynamic SpineCor orthosis for juvenile idiopathic scoliosis as well as to evaluate the stability of the spine after the weaning point.

**Material and Methods:**

For this study, 150 juvenile patients were treated by the SpineCor orthosis between 1993 and 2009. Of these, 67 patients had a definite outcome and 83 are still actively being treated. To determine the effectiveness of the brace the **OUTCOME **criteria recommended by the SRS was used.

**Results:**

The results from our study showed that of the 67 patients with a definite outcome, 32.9% corrected their Cobb angle by at least 5° and 10.5% had a stabilization of their Cobb angle. Within the patients with a definite outcome, 37.3% of patients where recommended for surgery before authorized end of treatment. For this group of patients, surgery was postponed. Looking at the stability of the curves 2 years after the end of the treatment, we found 12.5% of the patients continued their correction without the brace being used and 71.4% remained stable.

**Discussion:**

**From our study we can clearly see that the effectiveness of the SpineCor orthosis in obtaining and maintaining the neuromuscular integration of the corrective movement can be achieved effectively for juvenile patients**. Over 75% of all patients that finished the treatment had remained stable with a few continuing to correct their Cobb angle after the use of the SpineCor orthosis was discontinued.

**Conclusion:**

Our conclusion from this study is that the SpineCor orthosis is a very effective method of treatment of juvenile idiopathic scoliosis. The results obtained also indicate that treatment outcomes are better with early bracing. Most encouraging perhaps is the fact that the positive outcome appears to be maintained in the long term, and that surgery can be avoided or at least postponed.

## Introduction

Despite the great progresses made over the past years in the treatment of idiopathic scoliosis, little has been reported of juvenile idiopathic scoliosis as an entity and all the previous publications have contradicting results with respect to the sex incidence, types of curves and treatment results [1].

Juvenile idiopathic scoliosis is classically defined as scoliosis that is first diagnosed between the ages of 4 and 10. Some authors even divide this period in early onset juvenile scoliosis (under six years) and late onset juvenile scoliosis (over six and less than ten years old). Juvenile idiopathic scoliosis comprises about 10-15% of all idiopathic scoliosis in children [2]. Some authors found that boys are affected more at the younger end of the age spectrum and with more girls being affected at the upper end of the age spectrum [3]. Others reported an equal distribution [4,5]. Pehrsson and Nachemson [6] described the sex predilection divided by several periods: the female-to-male ratio is 1:1 between 4 and 6 years, 2:1 to 4:1 between six and less then 10 years old and can be 8:1 by the time the children are ten years of age, witch is a close ratio to the one reported for the adolescent idiopathic scoliosis [3.6.7]. It is suggested that patients with a curve of 20 degrees or more, particularly with a proven progression or a family history of scoliosis, should be treated rather than observed [8]. Juvenile curves of 30 degrees and more tend to continue to worsen without treatment and nearly 95% will require a surgical treatment [2].

Juvenile scoliosis remains one of the most challenging pediatric spine disorders.

Historic data have shown that untreated curves have the potential for serious cardiopulmonary and skeletal complications and even death [6].

Many conservative treatments are available for juvenile idiopathic scoliosis. Observation, casting, orthotic use, traction and surgical treatment are a few of the different options for managing juvenile idiopathic scoliosis. Surgical treatment should only be considered for patients who do not meet the criteria for observation or orthotic treatment or for those of them that have been unsuccessfully treated with either conservatory treatment. Although new fusion-less techniques may provide a treatment solution for a growing patient, it nonetheless poses a risk as with any surgery and so should be at least postponed as long as possible.

Current literature suggest that observation is still the first treatment for all small curves (<20 degrees) and for curves over 25 degrees some treatment should be considered due to the high probability of progression. Treatment should be considered earlier if the patient has a proven progression and an important family history of scoliosis. The main form of the conservative treatment still remains the brace, which was demonstrated to provide a reduction of curve progression, a decrease in the need for surgery and sometimes a correction of the existing deformity. In younger children who have a progressive deformity the preferred treatment is a Risser cast and for patients who have a progressive deformity but they are not candidates for bracing and casting, halo-gravity traction or fusion less technique are the methods to achieve deformity correction and improve respiratory mechanics.

Several types of braces have been used with varying degrees of success. These can be divided into two main categories depending on their mechanism of action: firstly, the rigid braces (following the three point pressure system, with derotation like PASB [9] or without derotation) and secondly, the SpineCor bracing system using the Corrective Movement^© ^principle.

The effectiveness of the SpineCor orthosis compared with the natural history of the disease has already been demonstrated for milder and moderate curves in patients with adolescent idiopathic scoliosis [10,11]. On these patients, the positive outcomes are maintained after skeletal maturity. The purpose of this study is to demonstrate, the already proven effectiveness of the SpineCor orthosis for AIS, on patients with juvenile idiopathic scoliosis.

## Materials and methods

### The studied population

The purpose of this prospective interventional study was to evaluate the effectiveness of the Dynamic SpineCor orthosis for juvenile idiopathic scoliosis and to evaluate the stability of the spine at 2 years after the skeletal maturity (weaning point of the orthosis). This study was carried out on a group of 150 patients (125 females and 25 males) having idiopathic scoliosis treated with the SpineCor orthosis.

Skeletal maturity is considered achieved when Risser 4 or more is reached. The United States grading system for Risser sign was used in this study.

Of the 150 patient cohort, 67 (60 females and 7 males) had an average Cobb angle at the beginning of treatment of 23° with a minimum of 15° and a maximum of 47° and a Standard Deviation of 7.2. These 67 patients had a definite outcome. The remaining 83 are still actively being treated. The assessment of the orthosis effectiveness was done following the **outcome criteria **proposed by the Scoliosis Research Society Committee on Bracing and Nonoperative Management ^12^.

**Figure 1:**
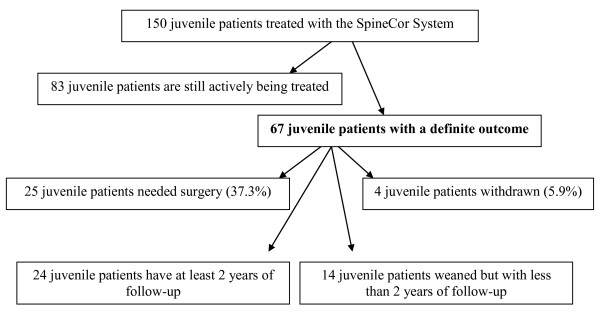
**The studied population**.

Twenty-four patients finished the treatment with the SpineCor orthosis **and had at least 2 years of follow-up**, 28 immature patients needed surgery (25 before the end of the treatment and 3 patients in the follow up period) and 4 patients withdrawn from the treatment. Fourteen patients have finished the treatment and have less than two years of follow up. (Figure 1)

All patients **regardless of the treatment compliance **have been included in the study.

### Radiographic analysis

For the initial pre-treatment radiograph a digital technique where the irradiation is half as much as that of a standard radiographs was used. The initial evaluation included standing postero-anterior and lateral radiographs without orthosis taken within one month prior to orthosis fitting. Control radiographs (standing PA) with the SpineCor orthosis (and shoe lift when prescribed) were taken on the day of the fitting, at 4-6 weeks and then every 5-6 months until weaning. Standing lateral radiographs were taken once a year. At the end of the treatment, the controls were continued at 6 months, one year and once every year. These evaluations were performed without orthosis. At the weaning point the patients are instructed to take off their brace at least 72 hours prior the X-ray.

### Description of the SpineCor System and treatment protocol

The Dynamic SpineCor orthosis, developed in 1992-93, uses a specific Corrective Movement^© ^which is dependant of the type of the curve. Curve classification was based on the classification of Leroux and Coillard [10]. For the treatment, the curve specific Corrective Movement^© ^is performed and the orthosis is applied according to definitions contained in the SpineCor Assistant Software. All the health providers need to complete a two-phase training course before fitting the SpineCor orthosis. The first phase involves reviewing of all information necessary to understand the "Corrective Movement Principle", the specific classifications and a workshop to fit the orthosis following these different corrective movements. The second phase consists of fitting the brace at their own practice of at least three or four patients with the help of a recognized trainer.

**Figure 2:**
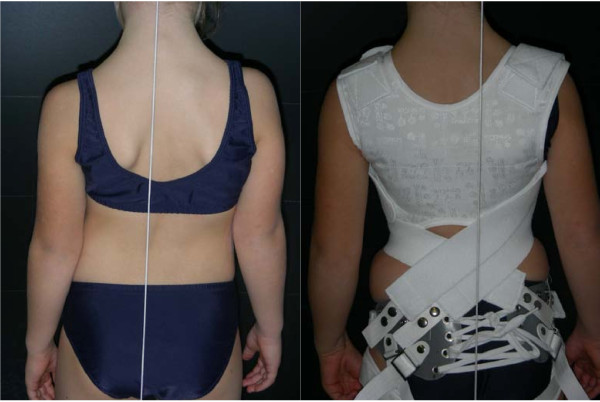
**Juvenile patient before and after the fitting of the SpineCor orthosis**.

In order to be effective and to obtain a neuromuscular integration the orthosis must maintain and amplify the corrective movement over time. Additionally, the orthosis must be worn 20 hours a day for a minimum of 18 months to create a neuromuscular integration of the Corrective Movement^© ^through active bio-feedback (Figure 2). Generally, the orthosis is stopped at skeletal maturity (at least Risser 4).

### Inclusion criteria

To be included in this study, the patients were already diagnosed with juvenile idiopathic scoliosis and had radiological confirmation of absence of significant pathological malformation of the spine at age over 4 years old but less than 10 when orthosis is prescribed and a Risser sign of 0. The initial Cobb angle of the patients needed to be equal to or above 15° with a proven progression and a family history of scoliosis. All patients in the study had no prior treatment for scoliosis.

### Exclusion criteria

The presence of a congenital malformation of the spine, spina bifida aperta, spondylolisthesis, neuromuscular scoliosis or postural scoliosis was considered as non-eligible for this study.

### Assessment of orthosis effectiveness

Improvement of more than 5° or stabilization of ±5° of the scoliosis curvature was defined as a positive outcome. The data collected were analyzed in four **outcomes **as suggested by the SRS Committee on Bracing and Non-operative Management [12]: 1) Percentage of patients who have 5° or less curve progression and the percentage of patients who have 6 degrees or more progression at skeletal maturity; 2) Percentage of patient who have had surgery recommendation/undergone before skeletal maturity; 3) Percentage of patients with curves exceeding 45° at maturity; 4) 2-years follow-up beyond skeletal maturity for each patient who was "successfully" treated with a brace to determine the percentage of patients who subsequently undergo surgery.

In order to compare and combine results with other studies, we stratified our results by curve type, curve magnitude grouping. The same stratification was used in order to compare the follow-up results. Descriptive statistics were employed to analyze the population.

All patients **regardless of the treatment compliance **have been included in the study.

## Results

### Assessment of orthosis effectiveness includes all of the following

#### 1. Percentage of patients who have 5° or less curve progression and the percentage of patients who have 6° or more progression at skeletal maturity

Twenty-nine of 67 patients (43.4%) corrected or stabilized their initial Cobb angle, and 9 patients (13.4%) had 6° or more progression of their initial Cobb angle (without surgery). From these 67 patients (Table 1), 24 reached the 2 years follow up (Table 2, 3).

**Table 1:** Outcome for the 67 juvenile patients treated by the SpineCor orthosis comparing the initial Cobb angle to the one at skeletal maturity (the weaning point).

SpineCor Dynamic Corrective Orthosis (n = 67)						
	**≤5°**	**>5°**	**(>45°)**	**Withdraw**	**Surgery**	**Total**

**Patients**	29	9	(2)	4	25	67

**Type of Curve**						
Thoracic	8	7	(2)	2	**18**	35
Thoracolumbar	17	1	-	1	3	22
Double	2	1	-	1	4	8
Lumbar	2	-	-	-	-	2

**Initial Cobb Angle**						
[<25 °]	20	6	(1)	2	10	38
[>26°]	9	3	(1)	2	**15**	29

**Table 2:** Outcome for the 24 juvenile patients treated by the SpineCor orthosis, comparing the Cobb angle at skeletal maturity (weaning point) to the one at 2 years follow-up post-bracing.

SpineCor Dynamic Corrective Orthosis (n = 24)					
	**≤5°**	(continuing correction)***	**>5°**	>45°*	Surgery**

**Patients (n)**	18	(3)	3	(3)	3

**Type of Curve**					
Thoracic	4	(2)	2	(1)	1
Thoracolumbar	10	(1)	1	(1)	1
Double	2	(-)	-	(1)	1
Lumbar	2	(-)	-	-	-

**Initial Cobb Angle**					
[<25°]	13	(2)	2	(2)	2
[>26°]	5	(1)	1	(1)	1

* Measured at 2 years of follow-up after skeletal maturity.

**Three patients undergone surgical treatment after the weaning of the orthosis and before the 2 years follow up mark.

*** 3 (12.5%) patients still improved from the time the orthosis was discontinued up to 2 years follow-up.

**Table 3:** Mean Cobb angle at the beginning of the treatment, weaning point and 2 years follow-up.

	Beginning of the treatment	Weaning point	2 years follow-up
**All juvenile patients** **(n = 150)**	**26.3°** (st dev 9.6)		

**Patients with definite outcome** **(n = 67)**	**28.1°** (st dev 10.7)	**20.7°** (st dev 15.4)	

**Patients with 2 years follow-up** **(n = 24)**	**23°** (st dev 6.9)	**13.1°** (st dev 12.3)	**13.1°** (st dev 12.8)

With post-orthosis treatment follow-up observation (Table 2), the treatment success rate (stabilization or correction of the curve) at 2 years was 75% (18/24), comparing the Cobb angle at skeletal maturity to the one at 2 years post skeletal maturity. Fifteen (62.5%) patients out of 24 stabilized their Cobb angle and 3 (12.5%) patients still improved from the time the orthosis was discontinued up to 2 years follow-up.

#### 2. Percentage of patient who have had surgery recommendation/undergone before skeletal maturity

Twenty-five immature patients out of 67 (37.3%) required surgical fusion while receiving treatment (Table 1). The average curve magnitude at the beginning of the treatment in this particular group was 34.9 ± 11.2° (range: 20-47°). General indication for fusion in all patients was progression of primary curve of more than 60° in thoracic region and 45° in thoracolumbar and lumbar region with trunk shift.

#### 3. Percentage of patients with curves exceeding 45° at maturity

In addition to patients referred for surgery before maturity, 2 patients out of 67 (2.9%) progressed beyond 45° at maturity (end of bracing Cobb angle) at 55° and 53° respectively (Table 1).

#### 4. 2-years follow-up beyond maturity for each patient who was "successfully" treated with a brace to determine the percentage of patients who subsequently undergo surgery

Twenty-four patients (22 girls and 2 boys) all treated by the SpineCor orthosis had at least 2 years of follow-up after skeletal maturity (Table 2).

Three of the 24 patients had curves exceeding 45° at 2 years follow-up and another three of them had a progression of their Cobb angle after skeletal maturity. Three patients had surgery recommendation after the end of the brace treatment in the two years follow up period.

#### 5. Results stratified by curve type and curve magnitude grouping at the weaning point (end of the treatment)

The results were analyzed separately by curve type (thoracic, thoracolumbar, lumbar, and double curves), curve magnitude, and skeletal maturity (Table 1 and 3) and reported to the 67 patients. The success of the orthotic treatment depending on curve type (Table 1) was achieved in 22.9% for thoracic [8/35], 77.3% for thoraco-lumbar [17/22], 25% for double [2/8] and 100% for lumbar curve [2/2] comparing the initial Cobb angle to the one at maturity. To study the effect of curve magnitude on outcome, the patients were divided into two groups. Group 1 consisted of 38 patients whose curves magnitude at the beginning of the treatment was less than 25°, and group 2 consisted of 29 patients with curve magnitude of 26° and higher. Group 1 had 52.7% [20/38] of success compared to 31.1% [9/29] of success for group 2.

#### 6. Follow-up results stratified by curve type and curve magnitude grouping

To quantify the success of treatment and the effectiveness of the orthosis we compared the results at the skeletal maturity (weaning point) as well at the 2 years follow-up. The results were analyzed separately by curve type (thoracic, thoracolumbar, lumbar, and double curves), and curve magnitude (Table 2). The results showed that correction was achieved even after the treatment was stopped in 28.5% [2/7] for thoracic and 8.3% [1/12] for thoraco-lumbar curves comparing the Cobb angle at skeletal maturity to the one at 2 years follow-up. To study the effect of curve magnitude on outcome, the patients were divided into two groups. Group 1 consisted of 17 patients whose curves magnitude at the beginning of the treatment was less than 25°, and group 2 consisted of 7 patients with curve magnitude of 26° and higher. Group 1 had 76.5% [13/17] of success and still corrected in 11.8% [2/17] of patients compared to 57.2% [4/7] of success and 14.3% [1/7] of continuing correction for group 2. Three patients underwent surgical treatment after skeletal maturity and before the two years follow-up.

## Discussion

The decision to begin orthotic treatment for idiopathic scoliosis (juvenile or adolescent) is a complex process and often not necessarily detached in term of the psychosocial and body image concerns for many patients and their families. It is therefore crucial that any treatment decision should be based on the best evidence available with respect to the effectiveness of the orthotic treatment, the rate of surgery (with and without treatment) and finally the patients own characteristics (Cobb angle, curve type, age of onset) as well as their specific risk factors [13,14].

Previous studies reported the effectiveness of the SpineCor orthosis in 2003 in the European Spine Journal on the first 195 patients and in 2007 in The Journal of Pediatric Orthopedics on a group of 493 patients suffering from adolescent idiopathic scoliosis. These studies indicate that the results are comparable for the juvenile patients. The preliminary study [10] in 2003 showed that on the 29 patients who had a minimum post-treatment follow-up of 2 years, 55% obtained a correction of their initial Cobb angle, 38% stabilized their Cobb angle and only 7% worsened by more than 5°. More recent results obtained also follow a similar trend. Comparing the Cobb angle at the end of the orthotic treatment to the one at 2 years post weaning point, the second study [11] showed that in 47 patients, that the follow-up of orthopedic treatment was a success in 95.7% of the patients with a mean correction of 8.6 ± 1.7°. Up to 33% of patients still correct their Cobb angle 5 years after the brace treatment is stopped. **The continuous correction can be explained by the capacity of the SpineCor orthosis to create a neuromuscular integration of the Corrective Movement^© ^through active bio-feedback**.

Most studies that use rigid brace systems show a slow loss of correction from the fitting point until the end of the treatment (when the curve is similar to the beginning of the treatment) and this is followed by an aggravation after the weaning point [15]. Several studies have also identified a trend of decreasing brace efficacy with increasing curve size and with an early onset. **There are very few publications that address the conservative treatment of idiopathic scoliosis with reference to outcome in juvenile patients**.

In 1992 Nachemson et al [6], in a long-term follow up study of patients with untreated scoliosis, concluded that there is a significantly increased mortality in infantile and juvenile scoliosis. This study also reported that there was an increased risk for death, which is related to juvenile scoliosis when the influence of the severity of the curve was taken into account. Previous reports confirm the same poor prognosis in early onset juvenile scoliosis; Branthwaite [16] described respiratory failure in idiopathic scoliosis with an onset before the age of 5. In our cohort only two patients had an early onset juvenile scoliosis (<6 years of age), one of them had surgical recommendation before skeletal maturity.

The unique characteristic of juvenile scoliosis is the tendency toward progression during the growth plateau (5-10 years) and rather than a rapid progression during the growth spurt. Due to this tendency of continuous progression, the natural course of juvenile idiopathic scoliosis is much more aggressive than that of adolescent idiopathic scoliosis. Between 70 and 95% of curves among patients with juvenile scoliosis progress and necessitate treatment; about half of this patients will need surgery [1,17,18]. Knowing these facts, the most important objective in bracing juvenile patients is to stop the progression to the point where surgery becomes the only option to improve the curve or maintain an acceptable level of cosmesis. For the juvenile patients that will have curve progression despite orthotic treatment the goal becomes different - the orthosis is then used to slow progression of a curve so surgery can be delayed as much as possible until more growth has been completed. The present study demonstrated that using the SpineCor orthosis can slow the progression of the curve and that the surgery could be delayed. Bunge et al [19] concluded that scoliosis patient and their families are prepared to undergo orthotic treatments only if it provides sizeable reduction of the risk of surgery and effectiveness and discomfort in wearing an orthosis were the most important determinants of the choices. All these results are important only if the effectiveness of the orthosis is demonstrated compared with the natural history of the disease.

The reported success of orthotic programs in the management of the juvenile idiopathic scoliosis is variable between the different authors and it seems to be centered on slowing/stopping the progression of the curve and to avoid or delay the spine fusion. Kahanovitz et al [20] reported an excellent prognosis with part time bracing for smaller curves and a poor prognosis for patients with higher Cobb angles (all patients needed surgery in this group). Tolo and Gillespie [7] found that only 27.2% (16/59) of their patients treated with the Milwaukee brace needed surgery and Dabney and Browen [21] had 33% surgery recommendations. Other authors have reported much higher percentages of patients who needed surgery despite bracing: Figueiredo and James [1] (modified Milwaukee brace) reported 62%, Manherz et al [18] reported 80% and finally McMaster 86% [22].

Comparing our results in this study to this reported above and to the expected progression rate of the juvenile idiopathic scoliosis (70-95%), we found that 25 immature patients out of 67 (37.3%) treated with the SpineCor orthosis required surgical fusion while receiving treatment. We also see that the percentage changes depending on the amplitude of the Cobb angle: 26.3% of the patients with curves under 25 degrees eventually needed surgery while 51.8% of the second group (>25°) had surgery recommended.

The results obtained in our study clearly show that the SpineCor System can alter the natural history of the Juvenile idiopathic scoliosis.

However, a limitation of the present study is that the results are based on patients treated with the SpineCor orthosis and are not compared with a non-treated control group or those treated by another type of brace (e.g. a rigid brace system). A more direct comparison with these two groups would provide a stronger basis for evaluating the efficacy of the SpineCor orthosis. At this stage however, very few studies have been published regarding the outcome of the conservative treatment for juvenile idiopathic scoliosis. Another limitation of the present study is that the proportion of thoracic/thoraco-lumbar/double curves in this study does not reflect the real proportion seen in the general population.

## Conclusion

This prospective study shows that SpineCor orthosis is an effective mode for the treatment of Juvenile idiopathic scoliosis and reveals a positive treatment outcome in the long run. The orthosis appears to be effective for milder curves (<25 degrees) as well as moderate curves (26-45 degrees) compared to the natural history of the disease. Moreover, the effectiveness of the orthotic treatment is demonstrated by its capacity to delay the surgical treatment. Among those who completed the course of treatment with the orthosis, the correction appears to be maintained at the long term because 75% of patients stabilized their Cobb angle and 12.5% patients still improved from the time the orthosis was discontinued up to 2 years follow-up.

## Competing interests

Charles H Rivard and Christine Coillard are consultants to Spinecorporation Ltd.

Alin Circo has no competing interests.

## Authors' contributions

All authors contributed equally to this work; all authors read and approved the final manuscript.

## Consent statement

Written informed consent was obtained from the patients or their parents/legal guardians for publication of this case report and accompanying images.
